# Gene and function diversity-area relationships in the inflammatory bowel disease fecal and mucosal microbiome

**DOI:** 10.3389/fmicb.2025.1660973

**Published:** 2026-01-06

**Authors:** Fubing Yu, Jiyang Song, Linyi Qi, Jinghua Liu, Yintiantian Yang, Wendy Li, Lianwei Li, Zhanshan (Sam) Ma

**Affiliations:** 1 Department of Gastroenterology, Affiliated Hospital of Yunnan University, Kunming, China; 2 Department of Cardiology, Gansu Provincial Hospital, Lanzhou, Gansu, China; 3 Institute of Medical Biology, Chinese Academy of Medical Sciences, Kunming, China; 4 College of Biological Sciences and Technology, Taiyuan Normal University, Jinzhong, China; 5 Microbiome Medicine and Advanced AI Technology, Kunming, China; 6 Computational Biology and Medical Ecology Lab, Kunming Institute of Zoology, Chinese Academy of Sciences, Kunming, China; 7 Biostatistics and Image Genetics Lab, Kunming Institute of Zoology, Chinese Academy of Sciences, Kunming, China

**Keywords:** inflammatory bowel disease (IBD), diversity-area relationship (DAR), metagenomic genes (MGs), metagenomic functional gene clusters (MFGCs), maximal accrual diversity (MAD)

## Abstract

The diversity-area relationship (DAR), an extension of the classic species-area relationship (SAR), provides a powerful framework for understanding how biodiversity scales across space. In this study, we applied DAR and its metagenomic counterpart (m-DAR) to investigate the spatial scaling of metagenomic genes (MGs) and metagenomic functional gene clusters (MFGCs) of seven functional databases in the gut microbiomes of individuals with inflammatory bowel disease (IBD) and healthy cohorts. Using shotgun sequencing data from 42 mucosal and 22 fecal samples from both healthy and IBD cohorts, we modeled how this MGs and MFGCs accrues with area (samples), estimating diversity scaling parameters (*z*), pair-wise diversity overlap (PDO), and maximal accrual diversity (MAD), which reflects the total potential diversity. We found that mucosal communities exhibited greater dissimilarity (less pair-wise diversity overlap) between individuals than fecal cowmmunities at the levels of gene richness and evenness (*q* = 1, 2), whereas fecal communities showed a stronger influence from dominant, abundant genes (*q* = 2, 3). Furthermore, healthy gut microbiomes showed greater similarity than those of IBD at the level of gene richness (*q* = 0), but showed greater dissimilarity at the level of abundant genes and dominant genes. Healthy gut microbiomes generally demonstrated a higher potential total diversity compared to those from IBD patients. Notably, fecal samples captured a broader range of microbial diversity than mucosal samples. Additionally, mucosal communities showed greater dissimilarity than fecal communities in almost all the MFGCs of the seven databases except ARDB, which showed the same trend as MGs. We also identified that specific functional clusters related to antibiotic resistance, such as genes for chloramphenicol and vancomycin resistance, displayed distinct scaling behaviors, suggesting their potential role in IBD pathogenesis. These findings demonstrate that the gut microbiome in IBD is not merely less diverse but is fundamentally restructured in its spatial architecture. The application of DAR provides a novel, quantitative insight to diagnose and understand this dysbiosis, moving beyond simple diversity metrics to capture the spatial diversity scaling of microbial genes and functions.

## Introduction

The term “*metagenome*” refers to the collective genetic material of microbial organisms within a given environment, which can be observed within or among individual “microbes” (single individuals or populations of individuals). Microbial biogeography describes the spatiotemporal distribution of microbial species, operational taxonomic units (OTUs; e.g., Martiny et al., 2006, Hanson et al., 2012, Costello et al., 2012, van der Gast, 2013), and, more broadly, the distribution of genes or metagenomes. These distributions can be studied both within and among individuals (Costello et al., 2012). OTUs, derived from amplicon sequencing reads, are commonly used to represent microorganisms at a taxonomic level. In contrast, operational metagenomic units (OMUs; Ma and Ellison, 2024, BMC Bioinformatics) are a recently proposed concept based on whole-genome sequencing (also known as shotgun sequencing) and represent metagenomic genes, providing a refined framework for analyzing functional diversity.

Our recent work has expanded the study of microbial diversity to include OTUs, genes, and metagenomes, with a focus on diversity-stability relationships and diseases linked to alterations in the structure and interactions of an individual’s microbiome. This research has been documented in a series of publications (Ma, 2018b, 2018c, 2019, 2020; Ma and Li, 2018; Ma et al., 2018; Ma and Ellison, 2018, 2019). Building on these efforts, we recently introduced the concept of operational metagenomic units (OMUs; Ma and Ellison, 2024, BMC Bioinformatics), which are based on the binning of sequencing reads from whole-genome metagenomic data. OMUs provide a refined framework for analyzing metagenomic diversity at the gene and functional levels. The diversity-area relationship (DAR; Ma, 2018a, 2018b, 2019), an extension of the classic species-area relationship (SAR; Connor and McCoy, 1979), is applicable to both OTUs and OMUs. In this study, we apply DAR to OTUs and its metagenomic counterpart, m-DAR, to OMUs, to investigate how microbial and metagenomic diversity fluctuate and scale across space.

Inflammatory bowel diseases (IBD), including Crohn’s disease (CD) and ulcerative colitis (UC), are thought to arise from an inappropriate immune response to gut microbes in genetically susceptible hosts. Despite extensive research, the aetiology of these chronic inflammatory disorders remains unclear. The incidence of IBD has risen significantly in the Western world since the mid-twentieth century (Molodecky et al., 2012; Rocchi et al., 2012; Hammer et al., 2016), with prevalence plateauing at up to 0.5% of the general population in developed nations. In contrast, IBD prevalence continues to increase in developing countries (Benchimol et al., 2009, 2014; Kaplan, 2015). Research into the causes of IBD has focused on host genetics, immune responses, gut microbiota, and environmental factors. A growing body of evidence highlights the consistent association between gut dysbiosis and IBD. Advances in high-throughput sequencing technologies over the past decade have further elucidated the role of the microbiome in IBD pathogenesis, revealing its functional mechanisms and underscoring its importance as a secondary organ system for the host.

In this study, we apply the DAR framework to investigate the scaling of microbial diversity (using OTUs) and metagenomic diversity (using OMUs) across space, expressed as Hill numbers (Hill, 1973; Chao et al., 2014). The DAR model (Ma et al., 2018; Ma and Ellison, 2021), an extension of the traditional Species-Area Relationship (SAR), provides a comprehensive framework for understanding how metagenomic diversity scales with the number of individuals sampled (Chen et al., 2021; Li L. W. and Ma, 2019; Li W. D. and Ma, 2019; Ma, 2018a, 2018b, 2018c, 2019; Ma and Ellison, 2024; Ma and Li, 2018; Ma et al., 2018; Xiao and Ma, 2021). For metagenomic data, we estimate m-DAR parameters and associated measures—pair-wise diversity overlap (PDO), maximal accrual diversity (MAD), and the ratio of individual- to population-level diversity (RIP)—to characterize the spatial scaling of metagenomic diversity within and among individuals. Using data from faecal and mucosal microbiomes of IBD and healthy cohorts, we illustrate our approach and analyze m-DAR profiles, PDO, and MAD to identify within- and among-individual variation and markers distinguishing healthy and IBD-associated microbiomes. This approach offers several key advantages: (1) It unifies alpha and beta diversity: The scaling parameter (z) effectively captures the rate of diversity turnover across space, integrating information often split between alpha and beta metrics. (2) It estimates total diversity potential: The model allows us to estimate the Maximal Accrual Diversity (MAD), which represents the total potential diversity of the ecosystem, including the “dark” diversity that is not yet observed but theoretically present. (3) It quantifies community overlap: It enables the calculation of Pair-wise Diversity Overlap (PDO) and the ratio of individual- to population-level diversity (RIP), direct measures of community similarity across scales.

This study holds significant implications for understanding microbial diversity in the context of IBD. First, by estimating diversity scaling rates across space for both microbial diversity (using OTUs) and functional diversity (using OMUs), we provide a quantitative framework to describe how microbial and metagenomic diversity change with scale, offering insights into the organization and distribution of microbial communities. Second, we estimate the potential (dark) diversity of genes and functionalities, which represents the portion of diversity not yet observed but theoretically present in the microbial community. This approach sheds light on the hidden diversity that may play critical roles in ecosystem functioning and disease states. Finally, we test the influence of IBD on diversity scaling and potential diversity, addressing how disease states alter the spatial organization and richness of microbial communities. These findings advance our understanding of the gut microbiome’s role in IBD and provide a foundation for future research on microbiome-based diagnostics and therapies.

## Materials and methods

### Study design and sample collection

A total of 22 faecal samples and 42 intestinal mucosal samples were collected from 21 couples, comprising 42 participants aged 18 to 60 years from Kunming, Yunnan, China. Healthy volunteers had no history of gastrointestinal disorders and had not used antibiotics in the year preceding sample collection or taken any medications during endoscopy. Ulcerative colitis (UC) diagnoses were confirmed using standard endoscopic, radiographic, and histopathological criteria. All UC patients were undergoing treatment with Mesalazine. Mucosal specimens were collected in the morning without prior bowel preparation. Samples were obtained 10 cm from the anal margin using disposable biopsy forceps, immediately flash-frozen in liquid nitrogen, and stored at −80 °C until DNA extraction. All data reanalyzed in this study are available in the publication by Li et al. (2021). Written and verbal informed consent was obtained from all participants.

### DNA extraction, sequencing, and preprocessing

Metagenomic shotgun sequencing was conducted on all 64 samples, yielding an average of 5.95 Gbp of high-quality data per sample. A non-redundant gene catalog comprising 999,310 genes was constructed. Sequencing was performed using the Illumina platform to generate paired-end reads. Raw reads were processed to remove low-quality sequences, reads containing ambiguous bases (N), and adapter sequences. For each sample, short reads were *de novo* assembled using multiple k-mer sizes in parallel. Assembled contigs were validated by mapping reads back to them, and the optimal assembly was selected based on contig N50 and mapping rate.

### Functional annotation

Gene functional annotation was performed by aligning sequences against several databases, including eggNOG, Nr, GO, COG, Swiss-Prot, KEGG, and ARDB. The Nr database (NCBI RefSeq Non-Redundant Protein Database) provides a comprehensive collection of non-redundant protein sequences. The eggNOG database (evolutionary genealogy of genes: Non-supervised Orthologous Groups) offers precise functional annotation through orthologous gene groups. KEGG (Kyoto Encyclopedia of Genes and Genomes) facilitates the interpretation of high-level biological functions and systems using molecular-level data. The Gene Ontology (GO) knowledgebase is a globally recognized resource for gene function information. COGs (Clusters of Orthologous Groups of proteins) enable the analysis of protein function and evolution. Swiss-Prot is a curated protein database containing detailed information on protein origin, sequence annotation, and amino acid sequences. ARDB (Antibiotic Resistance Genes Database) is a centralized resource for annotating antibiotic resistance genes.

### Estimation of metagenome diversity

Following the approach of Ma and Li (2018), metagenome diversity was estimated using Hill numbers (Hill, 1973; Jost, 2007; Chao et al., 2012, 2014) to quantify the diversity of metagenomes (MGs) and metagenomic functional gene clusters (MFGCs). The diversity of order *q* is defined as:


(1) $$D\;q={\left(\sum_{i=1}^{G}p_{i}^{q}\right)}^{1/(1-q)}$$

In Equation 1, *G* represents the number of MGs or MFGCs, *pᵢ* is the relative abundance of the *i*-th MG or MFGC, and *q* is the diversity order. For *q* = 0, ^0^*D* = *G* corresponds to the richness (number) of MGs or MFGCs. For *q* = 1, ^1^*D* represents diversity weighted by gene or functional gene cluster frequency. For *q* = 2, ^2^*D* emphasizes dominant (more abundant) genes or functional gene clusters, and for *q* = 3, ^3^*D* further increases the weighting of dominant genes or clusters (Ma and Li, 2018).

Metagenome diversity based on MGs relies on individual genes, whereas MFGC diversity can vary depending on whether clusters are defined by metabolic functions (e.g., KEGG) or protein functions (e.g., eggNOG). Ma and Li (2018) further classified MFGC diversity into two types based on gene abundance information. Type I MFGCs ignore gene abundances and count only the presence or absence of genes in a cluster (analogous to incidence data in macrobial diversity studies; e.g., Brom et al., 2015). In contrast, Type II MFGCs incorporate both gene presence and their relative abundances.

### Fitting *m*-DAR models and constructing *m*-DAR diversity profiles

Building on Ma (2018a), who extended the classic species-area relationship (SAR) to the diversity-area relationship (DAR), Ma and Ellison (2021) employed a power law (PL) model to define the metagenomic diversity-area relationship (m-DAR):


(2) $$D\;q=cA^{z}$$

In Equation 2, *^q^D* represents metagenome diversity of order *q* (Equation 1), *A* (“area”) denotes the number of sampled individuals, and *c* and *z* are fitted parameters. Here, *c* estimates the diversity of a single individual, while *z* represents the rate at which metagenome diversity increases with the number of individuals sampled. Following Plotkin et al. (2000) and Ulrich and Buszko (2003), Ma (2018a) modified Equation 2 to include a third parameter, *d*:


(3) $$D\;q=cA^{z}\exp(\mathrm{dA})$$

In this “power law with exponential cutoff” (PLEC) model, *d* < 0 and exp (*dA*) eventually overwhelms the exponential function at very large values of *A*, leading to an asymptotic value of *^q^D*. Using this exponential decay term makes sense because there are a finite number of people and thus a finite diversity of metagenomes. We use log-transformed versions of Equations 2, 3.


(4) ln(D)=ln(c)+zln(A)

(5) ln(D)=ln(c)+zln(A)+dA

To estimate the parameters of the models because their computation is simpler; *z* is scale-invariant in Equation 4; and the ecological interpretation of *z* as a scaling parameter is preserved in Equation 5. On a log–log plot, *z* is the slope of the linearized functions. Fitting of Equations 4, 5 to the data was evaluated using the linear correlation coefficients (*r*) and associated *p* values.

In these equations, *z* remains scale-invariant and retains its ecological interpretation as a scaling parameter. On a log–log plot, *z* represents the slope of the linearized functions. Model fitting was evaluated using linear correlation coefficients (*r*) and associated *p*-values. Unlike natural ecosystems, human microbiomes lack a natural spatial order or environmental gradient among hosts. To address this, we enumerated all possible permutations of sample subject orderings and randomly selected 50 (for MGs) or 100 (for MFGCs) orderings. For each permutation, m-DAR models (Equations 4, 5) were fitted. Poorly fitting models (*p* > 0.05) and PLEC models with biologically infeasible *A_max_* < 0 were excluded. The final model parameters were derived as averages from the remaining permutations. The relationship between diversity order *q* and scaling parameter *z* (Equation 2) defines the *m*-DAR profile, analogous to species diversity profiles (Ma, 2018a; Ma and Ellison, 2021).

### Metagenomic maximal accrual diversity of metagenome

Ma (2018*a*) derived the maximal accrual diversity (MAD) in a cohort or population based on the DAR-PLEC model (Equation 3) as:


(6) $$\;D\;q_{\max}=c{\left(-\frac{z}{d}\right)}^{z}\exp(-z)=cA_{\max}^{z}\exp(-z)$$

for which the number of individuals (*A*_max_) reaching the maximum diversity (*D*_max_) is estimated as:


(7) $$A_{\max}=-z/d$$

The m-MAD profile is defined as the set of *D_max_* values corresponding to different diversity orders *q*. *^q^D*_max_ serves as a proxy for potential (“dark”) diversity—genes or functional clusters absent locally but present in regional or global metagenomic pools (Partel et al., 2011; Real et al., 2017; Ma, 2019; Ma and Ellison, 2021).

### Pair-wise diversity overlap

Assuming equal “areas” for sampled individuals, the scaling parameter *z* from the basic m-DAR model (Equations 2, 4) was used to estimate pair-wise diversity overlap (PDO). The PDO, *g*, between two individuals (i.e., the proportion of new diversity in the second individual) is:


(8) $$g=2-2^{z}$$

Here, *g* ranges from 0 (no overlap; *z* = 1) to 1 (complete overlap; *z* = 0). The m-PDO profile is the set of *g* values corresponding to different diversity orders (*q*), approximating the similarity between pairs of human metagenomes.

## Results

### Metagenomic diversity-area relationships

**Figure 1:**
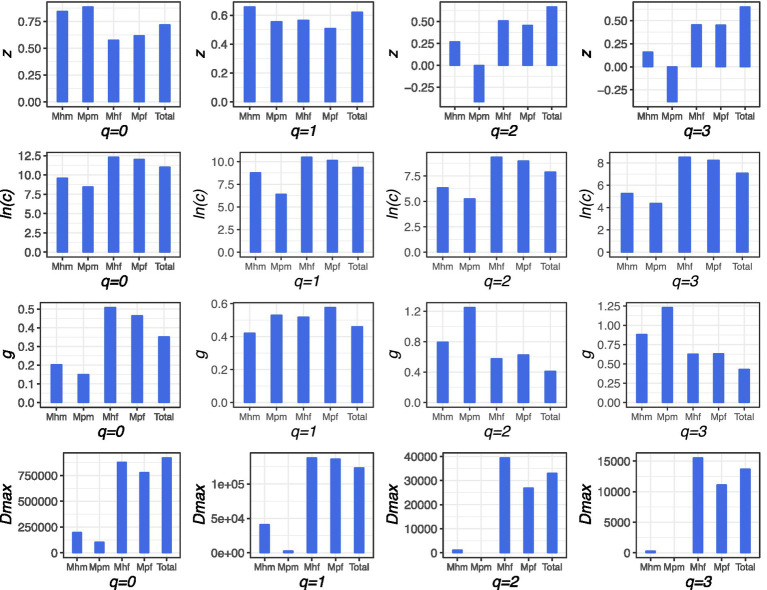
The scaling parameter (*z*), ln(*c*), *g*, and *D_max_* of the m-DAR (metagenome-diversity area relationship) for the metagenomic-genes (MGs) of the healthy cohorts, IBD cohorts, and the total samples. *Z* and ln(*c*) are model fitting parameters, *z* is the diversity scaling, *g* is pair-wise diversity overlap (PDO), and *D_max_* is maximal accrual diversity (MAD), which reflects the total potential diversity. Mhm is mucous microbiome of helahy cohorts, Mpm is mucous microbiome of IBD cohorts. Mhf is faecal microbiome of healthy cohorts and Mpf is faecal microbiome of IBD cohorts.

The basic *m*-DAR power law (PL) model (Equations 2, 4) and the PLEC model (Equations 3, 5) both demonstrated satisfactory fit to the faecal and mucous microbiome data (*p* < 0.05; Table 1; Supplementary Table S1; Figure 1). For diversity order *q* = 0 and *q* = 1, the scaling parameter (*z*) in Equation 2 for metagenomic genes (MGs) of mucous microbiome (Mhm and Mpm) was found to be larger than that of faecal microbiome (Mhf and Mpf). For example, the diversity order *q* = 0 yielded values of 0.845 and 0.887 for Mhm and Mpm, respectively, while the values for Mhf and Mpf were 0.576 and 0.618, respectively, the parameter of the total samples fell between these two values (0.720). Nevertheless, for diversity order *q* = 2 and *q* = 3, the *z* value of the mucous microbiome (Mhm and Mpm) is less than that of the faecal microbiome (Mhf and Mpf). Additionally, the *z* value of the total samples is greater than that of the aforementioned four split groups. From an alternative standpoint, the z-value of healthy cohorts (Mhm and Mhf) of mucous and faecal microbiome exhibits a lower value than that observed in IBD cohorts (Mpm and Mpf) in diversity order *q* = 0. However, in diversity orders *q* = 1, 2, and 3, this value is larger (with the exception of the negative value observed in Mpm in *q* = 2 and *q* = 3).

**Table 1:** The key parameters of m-DAR (metagenome diversity-area relationship) models fitted for metagenomic gene (MG) diversity, averaged from 100 times of re-sampling.

Orders	Treatments	*z*	ln(*c*)	*g*	*A_max_*	*D_max_*
*q* = 0	Mhm	0.845	9.603	0.203	36.3	198751.5
	Mhf	0.576	12.312	0.509	17.1	879345.6
	Mpm	0.887	8.460	0.150	65.4	104668.1
	Mpf	0.618	12.030	0.465	20.0	779333.9
	Total samples	0.720	11.020	0.352	49.0	922867.3
*q* = 1	Mhm	0.659	8.785	0.421	21.9	41185.0
	Mhf	0.566	10.505	0.519	16.1	138099.4
	Mpm	0.556	6.415	0.530	33.0	2838.2
	Mpf	0.509	10.146	0.577	58.6	136265.5
	Total samples	0.622	9.371	0.460	51.3	123460.4
*q* = 2	Mhm	0.270	6.347	0.794	17.1	1170.9
	Mhf	0.509	9.361	0.577	17.5	39485.9
	Mpm	−0.416	5.260	1.251	−6.8	NA
	Mpf	0.457	8.979	0.627	26.8	26916.4
	Total samples	0.668	7.899	0.411	42.4	33043.5
*q* = 3	Mhm	0.160	5.276	0.883	18.1	300.1
	Mhf	0.456	8.524	0.628	19.7	15563.2
	Mpm	−0.380	4.374	1.232	−9.5	NA
	Mpf	0.453	8.242	0.632	16.8	11129.4
	Total samples	0.650	7.086	0.431	40.8	13715.9

This pattern indicates fundamental differences in community organization. The higher *z*-values in mucosal samples at lower diversity orders (*q* = 0, 1) suggest that mucosal microbial communities are more dissimilar between individuals in terms of gene richness and the diversity of moderately abundant genes. In contrast, the higher *z*-values in faecal samples at higher diversity orders (q = 2, 3) point to a greater role of dominant, highly abundant genes in driving inter-individual differences in the luminal environment. The shift in *z*-values between healthy and IBD cohorts suggests that IBD is associated with increased variability in gene richness between individuals, but a more homogenized set of dominant genes.

As would be expected, the pair-wise diversity overlap (PDO) profile exhibited the opposite pattern of the *m-*DAR profile (parameter *g* in Table 1; Supplementary Table S1). This is because *z* in the *m-*DAR profile quantifies the dissimilarity of neighboring individuals whereas *g* in the PDO profile quantifies the overlap or similarity between individuals (Equation 8). The PLEC model indicated the existence of an asymptote for MG diversity (*d* < 0 and *D_max_* values presented in Table 1) (Equations 6 and 7), as well as the number of individual subjects (A_max_ values in Table 1; Supplementary Table S1) required to reach this asymptote. The maximal accrual diversity (MAD) of MGs in the gut metagenome is 9.2 × 10^5^ for the total samples of diversity order *q* = 0, which represents the estimated gene richness for this cohort. The faecal microbiome exhibits a larger MAD than the mucous microbiome for all four diversity orders (*q* = 0, 1, 2, 3; see Table 1; Figure 1). The magnitude of this difference is approximately four to seven times larger in the faecal microbiome than in the mucous microbiome (879,346 vs. 198,752 for Mhf vs. Mhm, 779,334 vs. 104,668 for Mpf vs. Mpm for diversity order q = 0). In comparing the healthy cohorts with the IBD cohorts, the former cohorts exhibited a larger MAD than the latter cohorts, with the exception of the NA value observed in Mpm in both q = 2 and q = 3.

The significantly higher MAD in faecal samples implies that the luminal microbiome possesses a much larger total potential diversity, or “gene pool,” than the mucosa-associated microbiome. This is consistent with the faecal microbiome representing a transient and diverse collection of microbes from throughout the gut, while the mucosal community is a more specialized, host-adapted subset. The generally greater MAD in healthy cohorts further suggests that a healthy state is associated with a greater reservoir of microbial genetic potential, which may be depleted in IBD.

### Metagenome functional gene cluster (MFGC) diversity-area relationships

**Figure 2:**
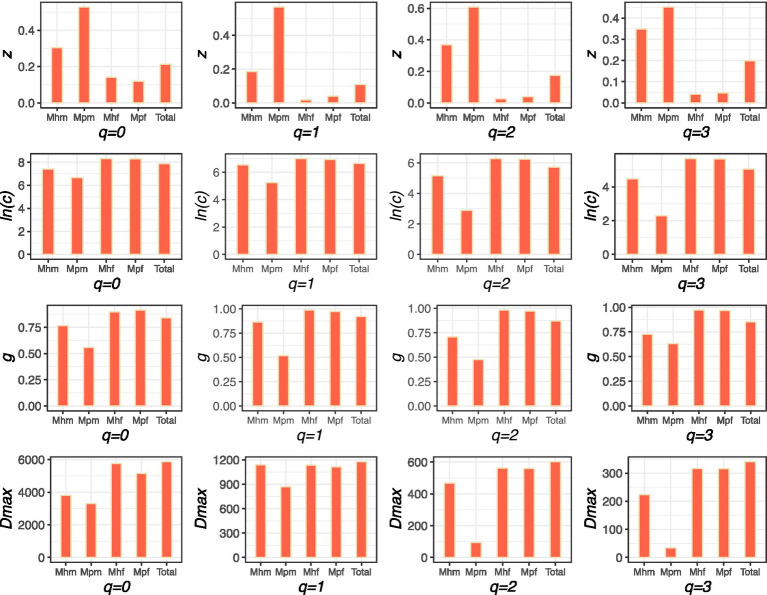
The scaling parameter (*z*), ln(*c*), *g*, and *D_max_* of the m-DAR (metagenome-diversity area relationship) for the MFGCs of KEGG database of the healthy cohorts, IBD cohorts and the total samples. *Z* and ln(*c*) are model fitting parameters, *z* is the diversity scaling, *g* is pair-wise diversity overlap (PDO), and *D_max_* is maximal accrual diversity (MAD), which reflects the total potential diversity. Mhm is mucous microbiome of heahty cohorts, Mpm is mucous microbiome of IBD cohorts. Mhf is faecal microbiome of healthy cohorts and Mpf is faecal microbiome of IBD cohorts.

The MFGC-DAR PL model was observed to accommodate all MFGC randomizations, as detailed in Supplementary Tables S2–S8. In general, the *z*-values for the mucous microbiome (Mhm and Mpm) are greater than those for the faecal microbiome (Mhf and Mpf) for diversity order q = 0 of MFGCs from all seven databases (Supplementary Tables S2–S8). For diversity orders q > 0, the *z*-value exhibited a similar pattern to that observed for q = 0 in the majority of MFGCs across all seven databases, with the exception of a few instances where negative values were observed, particularly for higher orders of Mpm. In the comparisons between healthy cohorts and IBD cohorts, the z-values of the healthy cohorts are lower than those of the IBD cohorts in the mucous microbiome across all seven databases, with the exception of instances where negative values were observed. However, this is not the case for the *z*-values of the faecal microbiome, which differ between databases (Supplementary Tables S2–S8). Nevertheless, the majority of z-values are observed to be larger in healthy cohorts than in IBD cohorts across all diversity orders (Figure 2).

As same patterns described above in *m*-DAR of MGs, the pair-wise diversity overlap (PDO) profile exhibited the opposite pattern of the *MFGC-*DAR profile (Supplementary Tables S2–S8). This is because *z* in the *MFGC-*DAR profile quantifies the dissimilarity of neighboring individuals whereas *g* in the PDO profile quantifies the overlap or similarity between individuals. As the PLEC mode of *m*-DAR, The PLEC model of MFGC-DAR also indicated the existence of an asymptote for MFGC diversity (*d* < 0 and *D_max_* values presented in Supplementary Tables S2–S8), as well as the number of individual subjects (A_max_ values in Supplementary Tables S2–S8) required to reach this asymptote. The faecal microbiome displays a greater MAD than the mucous microbiome across all four diversity orders (*q* = 0, 1, 2, 3) for seven databases, as illustrated in Supplementary Tables S2–S8. A comparison of the healthy cohorts with the IBD cohorts revealed that the former exhibited a larger MAD than the latter, with the exception of the NA value observed in Mpm. The same patterns are observed in *MFGC*-DAR with *m-*DAR models.

### Metagenomic genes of MFGCs diversity-area relationships

**Figure 3:**
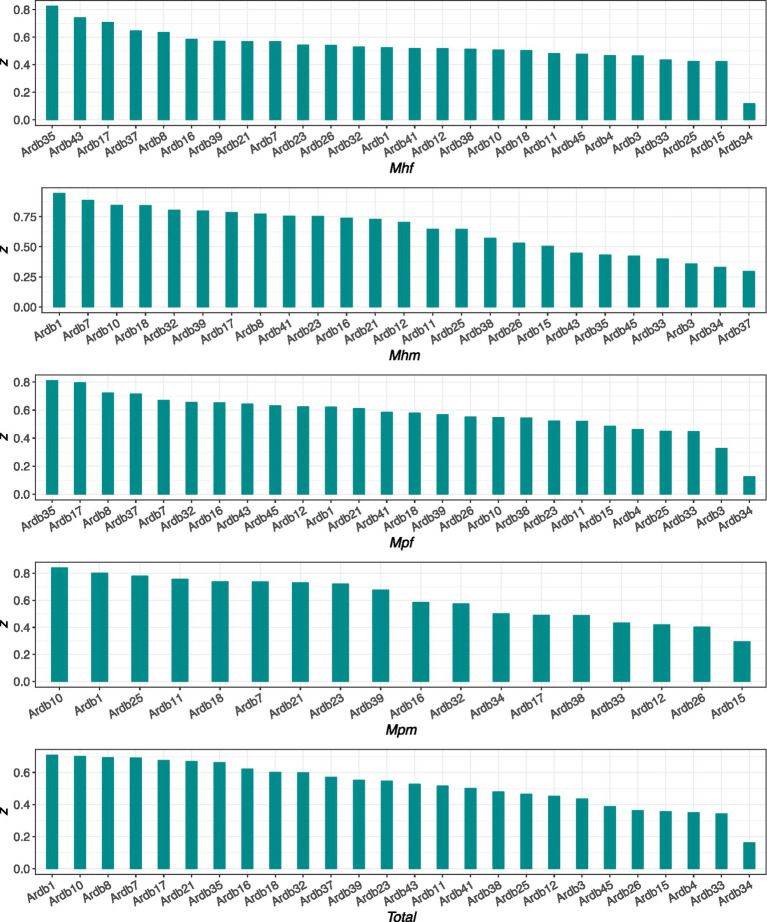
The scaling parameter (*z*) of the *MFGC*-DAR (metagenome-diversity area relationship) for the metagenomic-genes (MGs) of each ARDB MFGCs for the healthy cohorts, IBD cohorts, and the total samples, respectively.

We proceeded to fit DAR models with MGs of each MFGC (for the purposes of this example, we selected the MFGCs contained within the ARDB database, which included those with more than 10 MGs). Figure 3 illustrates the *z*-value of the DAR model for each MFGC, which is employed to facilitate the sorting of the MFGCs for each cohort. As illustrated in the figure, the MFGCs with the highest *z*-values in the faecal microbiome are ardb 35, ardb 43 and ardb 17 in Mhf, and ardb35, ardb 17 and ardb8 in Mpf. The MFGCs with the highest z-values in the mucous microbiome are ardb1, ardb7, and ardb10 in Mhm, and ardb10, ardb1, and ardb25 in Mpm. Ardb 35 is “Group B chloramphenicol acetyltransferase, which can inactivate chloramphenicol. Also referred to as xenobiotic acetyltransferase,” ardb 17 is “Virginiamycin A acetyltransferase, which can inactivate the target drug,” ardb 1 is “VanG type vancomycin resistance operon genes, which can synthesize peptidoglycan with modified C-terminal D-Ala-D-Ala to D-alanine--D-serine,” ardb 10 is “ABC transporter system, bacitracin efflux pump.” It is possible that the MFGC with different z-values may play a distinct role in the development of IBD.

The distinct scaling behaviors of specific antibiotic resistance genes (ARGs) are noteworthy. The top-scaling ARGs in faecal samples (ardb35: chloramphenicol acetyltransferase; ardb17: virginiamycin A acetyltransferase) and mucosal samples (ardb1: vancomycin resistance operon; ardb10: bacitracin efflux pump) suggest that these resistance mechanisms may play niche-specific and potentially important roles in shaping the microbial community structure in health and disease, possibly reflecting selective pressures within the gut ecosystem of IBD patients.

## Discussion

The application of the diversity-area relationship (DAR) and its metagenomic counterpart (m-DAR) to the gut microbiome has provided novel insights into the spatial scaling of microbial gene and functional diversity in both healthy individuals and those with inflammatory bowel disease (IBD). By extending the classic species-area relationship (SAR; Connor and McCoy, 1979) to metagenomic data, this study demonstrates how microbial diversity scales across individuals and highlights the distinct organizational patterns of the gut microbiome in health and disease. Our findings reveal significant differences in diversity scaling between faecal and mucosal microbiomes, as well as between healthy and IBD cohorts, underscoring the utility of DAR and m-DAR in characterizing microbial ecosystems (Ma, 2018a; Ma and Li, 2018).

One of the key findings of this study is the contrasting diversity scaling patterns between faecal and mucosal microbiomes. For metagenomic genes (MGs), the mucosal microbiome exhibited higher scaling parameters (*z*-values) than the faecal microbiome at diversity orders *q* = 0 and *q* = 1, indicating greater dissimilarity between individuals in mucosal communities at lower diversity orders. However, this trend reversed at *q* = 2 and *q* = 3, where the faecal microbiome showed higher *z*-values, suggesting a greater influence of dominant genes in faecal communities. These results align with the known ecological differences between mucosal and luminal (faecal) environments. The mucosal microbiome is shaped by host-derived factors such as immune responses and mucosal adhesion, leading to higher inter-individual variability at the level of gene richness (*q* = 0) and frequency-weighted diversity (*q* = 1). In contrast, the faecal microbiome, which is influenced by diet and transit time, tends to have a more stable core of dominant taxa, reflected in higher z-values at *q* = 2 and *q* = 3 (Ma, 2018a; Ma and Li, 2018).

The maximal accrual diversity (MAD) further highlighted these differences, with the faecal microbiome exhibiting significantly higher MAD than the mucosal microbiome across all diversity orders. This suggests that faecal samples capture a broader range of microbial diversity, likely due to their representation of transient luminal communities. In contrast, mucosal samples, while more variable between individuals, may reflect a more specialized and host-adapted subset of the microbiome. These findings have important implications for microbiome sampling strategies, as they underscore the complementary nature of faecal and mucosal samples in capturing different aspects of microbial diversity (Ma, 2018a; Ma et al., 2018).

The comparison between healthy and IBD cohorts revealed distinct scaling patterns that may reflect the underlying pathophysiology of IBD. At *q* = 0, IBD cohorts exhibited higher *z*-values than healthy cohorts, indicating greater inter-individual variability in gene richness between individuals within IBD. This finding aligns with the established concept of dysbiosis in IBD, where disease-associated changes lead to a less stable and more heterogeneous microbial community structure between patients (Frank et al., 2007; Gevers et al., 2014). However, at higher diversity orders (*q* = 1, 2, and 3), healthy cohorts showed higher *z*-values, suggesting that IBD cohorts have a more uniform distribution of dominant genes. This could reflect the loss of rare taxa and the expansion of dominant, potentially pathogenic species in IBD, a phenomenon previously observed in microbiome studies of IBD patients (Ma, 2018a; Ma and Ellison, 2019).

The MAD results further supported these observations, with healthy cohorts generally exhibiting higher MAD than IBD cohorts. This suggests that healthy microbiomes have a greater potential for accruing diversity, possibly due to the presence of a more balanced and resilient community. In contrast, the reduced MAD in IBD cohorts may reflect the loss of microbial diversity and functional redundancy associated with disease. These findings highlight the potential of DAR and m-DAR as tools for quantifying dysbiosis and monitoring disease progression in IBD (Ma, 2018a; Ma and Li, 2018).

The analysis of metagenomic functional gene clusters (MFGCs) provided additional insights into the functional organization of the gut microbiome. Similar to the patterns observed for MGs, mucosal microbiomes showed higher *z*-values than faecal microbiomes at *q* = 0, but this trend varied across functional databases and diversity orders. Notably, specific MFGCs associated with antibiotic resistance, such as chloramphenicol acetyltransferase (ardb35) and vancomycin resistance operon genes (ardb1), exhibited distinct scaling behaviors. These MFGCs were among the top contributors to diversity scaling in both faecal and mucosal microbiomes, suggesting their potential roles in shaping microbial community structure (Ma and Ellison, 2024; Ma and Li, 2018). The prominence of antibiotic resistance genes in IBD cohorts is particularly noteworthy, as it may reflect the selective pressures imposed by antibiotic use or the dysregulated immune responses characteristic of IBD. The distinct scaling behaviors of these MFGCs suggest that they may contribute to the functional dysbiosis observed in IBD, potentially influencing disease severity and treatment outcomes. Future studies could explore the functional consequences of these scaling patterns, such as their impact on microbial resilience and host–microbe interactions (Ma, 2018a; Ma and Ellison, 2019).

The application of DAR and m-DAR to metagenomic data represents a significant methodological advancement in microbiome research. By extending the SAR framework—a cornerstone of island biogeography and macroecology—to include gene and functional diversity, these models provide a unified approach for quantifying microbial diversity across spatial scales. The parameter *z*, which quantifies the rate of diversity accumulation, has a direct analog in ecological theory, where higher *z*-values are typically associated with communities in more heterogeneous environments. Our observation of higher *z*-values in mucosal samples at lower *q*-orders may therefore be interpreted as evidence of a more individualized and patchy ecological landscape at the mucosal interface, consistent with its role as a primary host–microbe interaction site. The use of Hill numbers and the PLEC model, which accounts for finite diversity, further enhances the ecological realism of this approach (Chao et al., 2014; Ma, 2018a).

The inclusion of the power law with exponential cutoff (PLEC) model adds another layer of ecological realism, as it accounts for the finite nature of microbial diversity in human populations. The estimation of maximal accrual diversity (MAD) and pair-wise diversity overlap (PDO) further enriches the analytical toolkit, providing measures of potential (“dark”) diversity and inter-individual similarity, respectively. These metrics have broad applicability beyond IBD, offering new ways to study microbial diversity in other diseases, environmental microbiomes, and host-associated ecosystems (Ma, 2018a; Ma and Li, 2018).

While this study demonstrates the utility of DAR and m-DAR in characterizing microbial diversity, several limitations should be acknowledged. First, the sample size, though sufficient for initial exploration, may limit the generalizability of the findings. Second, potential technical biases, including variation in sequencing depth across samples, the dependence of functional annotation on the choice of reference databases, and the inherent sampling heterogeneity between faecal and mucosal sites (e.g., differences in biomass and host DNA contamination), could influence diversity estimates and comparisons. Larger, multi-center cohorts and standardized protocols would help mitigate these issues. Third, the reliance on cross-sectional data limits causal inference. Longitudinal studies tracking changes in diversity scaling over time and in response to treatment are needed.

Future research should explore the integration of DAR and m-DAR with other omics data to provide a more holistic understanding of host–microbe interactions (Prins et al., 2024; Valdés-Mas et al., 2025; Zhang et al., 2025; Ning et al., 2023; Zhao et al., 2025). For instance, recent work has shown that circulating and tissue microRNAs, such as those identified by Tocia et al. (2023), reflect disease activity in Crohn’s disease. Combining such host-derived molecular biomarkers with our ecological scaling analysis could bridge the gap between microbial community structure and host response, significantly broadening the translational relevance of this approach. Applying these models to other microbial ecosystems could also reveal general principles of microbial diversity scaling.

In conclusion, this study demonstrates the power of DAR and m-DAR in characterizing the spatial scaling of microbial gene and functional diversity in the gut microbiome. By revealing distinct scaling patterns in faecal and mucosal microbiomes, as well as between healthy and IBD cohorts, these models provide new insights into the ecological organization of the gut microbiome and its role in health and disease. The identification of specific MFGCs associated with antibiotic resistance further highlights the potential of this approach for uncovering functional drivers of dysbiosis. As microbiome research continues to evolve, DAR and m-DAR offer promising tools for advancing our understanding of microbial ecosystems and their implications for human health (Ma, 2018a; Ma and Li, 2018).

## Data Availability

The original contributions presented in the study are included in the article/Supplementary material, further inquiries can be directed to the corresponding authors.
