# Supplementary material for: Gene and function diversity-area relationships in the inflammatory bowel disease fecal and mucosal microbiome
Source: Front Microbiol. 2026 Jan 6;16:1660973. doi: 10.3389/fmicb.2025.1660973 (PMC12815819; doi:10.3389/fmicb.2025.1660973)
Supplement: Supplementary file 1 [file Table_1.DOCX]

**Online Supplementary Tables S1-S7**

**Table S1**. The parameters of m-DAR (metagenome diversity-area relationship) models fitted for MFGC (metagenome functional gene cluster) diversity, averaged from 100 times of re-sampling (ARDB)

| **Orders** | **Treatments** | **m-DAR PL (power law) Model** | | | | | | **m-DAR PLEC (power law with exponential cutoff) Model** | | | | | | |
| --- | --- | --- | --- | --- | --- | --- | --- | --- | --- | --- | --- | --- | --- | --- |
|  |  | ***z*** | **ln(*c*)** | *g* | *R* | ***p*-value** | ***N*** | ***z*** | ***d*** | **ln(*c*)** | *R* | ***p*-value** | ***A_max_*** | ***D_max_*** |
| *q*=0 | Mhm | 0.241 | 3.005 | 0.818 | 0.915 | 0.000 | 21 | 0.457 | -0.031 | 2.875 | 0.963 | 0.000 | 14.936 | 38.607 |
|  | Mhf | 0.074 | 3.659 | 0.948 | 0.940 | 0.000 | 11 | 0.107 | -0.008 | 3.653 | 0.966 | 0.000 | 13.579 | 45.852 |
|  | Mpm | 0.432 | 2.241 | 0.651 | 0.906 | 0.000 | 21 | 0.798 | -0.052 | 2.021 | 0.954 | 0.000 | 15.404 | 30.108 |
|  | Mpf | 0.087 | 3.657 | 0.938 | 0.944 | 0.000 | 11 | 0.147 | -0.014 | 3.647 | 0.975 | 0.000 | 10.468 | 46.731 |
|  | Total Samples | 0.131 | 3.387 | 0.905 | 0.808 | 0.000 | 64 | 0.285 | -0.008 | 3.162 | 0.891 | 0.000 | 34.375 | 48.676 |
| *q*=1 | Mhm | 0.091 | 2.187 | 0.935 | 0.721 | 0.004 | 21 | 0.189 | -0.015 | 2.149 | 0.817 | 0.003 | 12.292 | 11.408 |
|  | Mhf | 0.068 | 2.177 | 0.952 | 0.783 | 0.011 | 11 | 0.088 | -0.008 | 2.190 | 0.871 | 0.009 | 11.120 | 10.104 |
|  | Mpm | 0.215 | 1.783 | 0.839 | 0.798 | 0.002 | 21 | 0.489 | -0.040 | 1.637 | 0.904 | 0.001 | 12.232 | 10.725 |
|  | Mpf | 0.085 | 2.012 | 0.939 | 0.845 | 0.006 | 11 | 0.085 | -0.004 | 2.042 | 0.907 | 0.005 | 22.863 | 9.226 |
|  | Total Samples | 0.048 | 2.091 | 0.966 | 0.615 | 0.004 | 64 | 0.083 | -0.002 | 2.062 | 0.674 | 0.002 | 35.955 | 9.733 |
| *q*=2 | Mhm | 0.029 | 1.703 | 0.980 | 0.692 | 0.005 | 21 | 0.060 | -0.005 | 1.696 | 0.785 | 0.004 | 11.249 | 5.939 |
|  | Mhf | 0.098 | 1.512 | 0.930 | 0.831 | 0.007 | 11 | 0.048 | 0.004 | 1.565 | 0.880 | 0.011 | -11.646 | NA |
|  | Mpm | 0.106 | 1.439 | 0.924 | 0.738 | 0.004 | 21 | 0.286 | -0.028 | 1.381 | 0.826 | 0.002 | 10.116 | 5.791 |
|  | Mpf | 0.124 | 1.296 | 0.910 | 0.810 | 0.007 | 11 | 0.083 | -0.001 | 1.384 | 0.877 | 0.009 | 75.077 | 5.266 |
|  | Total Samples | 0.037 | 1.516 | 0.974 | 0.563 | 0.004 | 64 | 0.037 | 0.000 | 1.528 | 0.658 | 0.002 | 108.731 | 5.292 |
| *q*=3 | Mhm | 0.027 | 1.466 | 0.981 | 0.690 | 0.007 | 21 | 0.049 | -0.004 | 1.469 | 0.796 | 0.004 | 11.574 | 4.664 |
|  | Mhf | 0.126 | 1.231 | 0.909 | 0.845 | 0.007 | 11 | 0.059 | 0.008 | 1.292 | 0.886 | 0.010 | -7.283 | NA |
|  | Mpm | 0.066 | 1.279 | 0.953 | 0.743 | 0.003 | 21 | 0.210 | -0.023 | 1.249 | 0.803 | 0.003 | 9.083 | 4.491 |
|  | Mpf | 0.129 | 1.060 | 0.906 | 0.772 | 0.011 | 11 | 0.110 | -0.005 | 1.142 | 0.868 | 0.009 | 20.360 | 3.912 |
|  | Total Samples | 0.044 | 1.269 | 0.969 | 0.572 | 0.005 | 64 | 0.030 | 0.000 | 1.308 | 0.654 | 0.003 | -84.975 | NA |

**Table S2**. The parameters of m-DAR (metagenome diversity-area relationship) models fitted for MFGC (metagenome functional gene cluster) diversity, averaged from 100 times of re-sampling (COG)

| **Orders** | **Treatments** | **m-DAR PL (power law) Model** | | | | | | **m-DAR PLEC (power law with exponential cutoff) Model** | | | | | | |
| --- | --- | --- | --- | --- | --- | --- | --- | --- | --- | --- | --- | --- | --- | --- |
|  |  | ***z*** | **ln(*c*)** | *g* | *R* | ***p*-value** | ***N*** | ***z*** | ***d*** | **ln(*c*)** | *R* | ***p*-value** | ***A_max_*** | ***D_max_*** |
| *q*=0 | Mhm | 0.293 | 6.942 | 0.775 | 0.940 | 0.000 | 21 | 0.495 | -0.029 | 6.820 | 0.971 | 0.000 | 17.3 | 2288.8 |
|  | Mhf | 0.125 | 7.805 | 0.910 | 0.951 | 0.000 | 11 | 0.167 | -0.010 | 7.798 | 0.974 | 0.000 | 16.6 | 3296.2 |
|  | Mpm | 0.453 | 6.369 | 0.631 | 0.931 | 0.000 | 21 | 0.763 | -0.044 | 6.182 | 0.966 | 0.000 | 17.4 | 1990.9 |
|  | Mpf | 0.101 | 7.797 | 0.928 | 0.952 | 0.000 | 11 | 0.172 | -0.017 | 7.785 | 0.982 | 0.000 | 10.3 | 3019.1 |
|  | Total Samples | 0.148 | 7.540 | 0.892 | 0.871 | 0.000 | 64 | 0.268 | -0.006 | 7.365 | 0.927 | 0.000 | 41.5 | 3284.5 |
| *q*=1 | Mhm | 0.113 | 6.423 | 0.918 | 0.810 | 0.001 | 21 | 0.270 | -0.022 | 6.331 | 0.906 | 0.000 | 12.0 | 839.7 |
|  | Mhf | 0.035 | 6.623 | 0.975 | 0.819 | 0.006 | 11 | 0.050 | -0.005 | 6.633 | 0.880 | 0.009 | 9.7 | 809.6 |
|  | Mpm | 0.261 | 6.091 | 0.802 | 0.861 | 0.000 | 21 | 0.566 | -0.044 | 5.912 | 0.935 | 0.000 | 13.0 | 895.8 |
|  | Mpf | 0.020 | 6.630 | 0.986 | 0.839 | 0.006 | 11 | 0.034 | -0.004 | 6.631 | 0.891 | 0.007 | 8.7 | 789.5 |
|  | Total Samples | 0.032 | 6.574 | 0.978 | 0.644 | 0.001 | 64 | 0.070 | -0.002 | 6.521 | 0.747 | 0.001 | 33.2 | 809.5 |
| *q*=2 | Mhm | 0.096 | 5.939 | 0.931 | 0.778 | 0.002 | 21 | 0.216 | -0.018 | 5.885 | 0.862 | 0.002 | 11.8 | 493.6 |
|  | Mhf | 0.043 | 6.092 | 0.970 | 0.798 | 0.008 | 11 | 0.066 | -0.008 | 6.106 | 0.877 | 0.010 | 8.4 | 483.5 |
|  | Mpm | 0.204 | 5.732 | 0.849 | 0.831 | 0.001 | 21 | 0.460 | -0.037 | 5.590 | 0.912 | 0.001 | 12.4 | 537.5 |
|  | Mpf | 0.021 | 6.113 | 0.985 | 0.821 | 0.007 | 11 | 0.040 | -0.005 | 6.112 | 0.892 | 0.007 | 8.5 | 472.5 |
|  | Total Samples | 0.030 | 6.064 | 0.979 | 0.612 | 0.001 | 64 | 0.056 | -0.002 | 6.032 | 0.713 | 0.000 | 35.9 | 481.9 |
| *q*=3 | Mhm | 0.099 | 5.556 | 0.929 | 0.773 | 0.002 | 21 | 0.216 | -0.018 | 5.507 | 0.847 | 0.002 | 11.9 | 339.1 |
|  | Mhf | 0.059 | 5.704 | 0.958 | 0.806 | 0.009 | 11 | 0.113 | -0.015 | 5.712 | 0.898 | 0.006 | 7.6 | 339.8 |
|  | Mpm | 0.180 | 5.422 | 0.867 | 0.811 | 0.001 | 21 | 0.406 | -0.032 | 5.293 | 0.897 | 0.001 | 12.5 | 369.4 |
|  | Mpf | 0.035 | 5.725 | 0.975 | 0.825 | 0.008 | 11 | 0.062 | -0.007 | 5.723 | 0.915 | 0.003 | 8.9 | 329.1 |
|  | Total Samples | 0.035 | 5.688 | 0.975 | 0.602 | 0.003 | 64 | 0.058 | -0.001 | 5.662 | 0.706 | 0.001 | 41.3 | 337.2 |

**Table S3**. The parameters of m-DAR (metagenome diversity-area relationship) models fitted for MFGC (metagenome functional gene cluster) diversity, averaged from 100 times of re-sampling (eggNOG)

| **Orders** | **Treatments** | **m-DAR PL (power law) Model** | | | | | | **m-DAR PLEC (power law with exponential cutoff) Model** | | | | | | |
| --- | --- | --- | --- | --- | --- | --- | --- | --- | --- | --- | --- | --- | --- | --- |
|  |  | ***z*** | **ln(*c*)** | *g* | *R* | ***p*-value** | ***N*** | ***z*** | ***d*** | **ln(*c*)** | *R* | ***p*-value** | ***A_max_*** | ***D_max_*** |
| *q*=0 | Mhm | 0.266 | 11.412 | 0.797 | 0.950 | 0.000 | 21 | 0.416 | -0.021 | 11.322 | 0.976 | 0.000 | 19.6 | 187992.3 |
|  | Mhf | 0.126 | 12.233 | 0.909 | 0.971 | 0.000 | 11 | 0.155 | -0.007 | 12.228 | 0.986 | 0.000 | 22.4 | 283374.4 |
|  | Mpm | 0.455 | 10.734 | 0.629 | 0.949 | 0.000 | 21 | 0.639 | -0.026 | 10.623 | 0.972 | 0.000 | 24.5 | 167350.5 |
|  | Mpf | 0.126 | 12.176 | 0.909 | 0.974 | 0.000 | 11 | 0.190 | -0.015 | 12.165 | 0.991 | 0.000 | 12.5 | 256604.6 |
|  | Total Samples | 0.213 | 11.784 | 0.841 | 0.875 | 0.000 | 64 | 0.376 | -0.009 | 11.547 | 0.929 | 0.000 | 43.1 | 292002.6 |
| *q*=1 | Mhm | 0.171 | 8.565 | 0.874 | 0.723 | 0.003 | 21 | 0.351 | -0.029 | 8.507 | 0.810 | 0.002 | 12.1 | 8350.8 |
|  | Mhf | 0.034 | 9.224 | 0.976 | 0.793 | 0.009 | 11 | 0.049 | -0.006 | 9.235 | 0.867 | 0.010 | 8.8 | 10860.6 |
|  | Mpm | -0.404 | 7.904 | 1.244 | 0.682 | 0.004 | 21 | 0.292 | -0.082 | 7.198 | 0.753 | 0.005 | 3.6 | 1447.5 |
|  | Mpf | 0.033 | 9.191 | 0.977 | 0.842 | 0.007 | 11 | 0.062 | -0.008 | 9.199 | 0.891 | 0.008 | 7.6 | 10540.2 |
|  | Total Samples | 0.149 | 8.755 | 0.891 | 0.651 | 0.001 | 64 | 0.359 | -0.012 | 8.461 | 0.797 | 0.000 | 30.9 | 11317.5 |
| *q*=2 | Mhm | 0.189 | 6.366 | 0.860 | 0.760 | 0.003 | 21 | 0.355 | -0.026 | 6.300 | 0.836 | 0.002 | 13.8 | 968.8 |
|  | Mhf | 0.030 | 7.686 | 0.979 | 0.783 | 0.011 | 11 | 0.085 | -0.014 | 7.692 | 0.853 | 0.013 | 6.0 | 2342.9 |
|  | Mpm | -0.619 | 5.867 | 1.349 | 0.679 | 0.007 | 21 | 0.165 | -0.092 | 5.130 | 0.777 | 0.003 | 1.8 | 157.9 |
|  | Mpf | 0.020 | 7.683 | 0.986 | 0.808 | 0.009 | 11 | 0.074 | -0.013 | 7.677 | 0.883 | 0.007 | 5.6 | 2275.4 |
|  | Total Samples | 0.216 | 6.969 | 0.838 | 0.593 | 0.003 | 64 | 0.487 | -0.016 | 6.625 | 0.750 | 0.000 | 30.6 | 2448.9 |
| *q*=3 | Mhm | 0.157 | 5.384 | 0.885 | 0.752 | 0.004 | 21 | 0.262 | -0.018 | 5.371 | 0.824 | 0.002 | 14.7 | 334.7 |
|  | Mhf | -0.003 | 6.857 | 1.002 | 0.796 | 0.009 | 11 | 0.032 | -0.006 | 6.826 | 0.852 | 0.013 | 5.4 | 941.4 |
|  | Mpm | -0.538 | 5.100 | 1.311 | 0.678 | 0.007 | 21 | 0.108 | -0.075 | 4.485 | 0.779 | 0.003 | 1.5 | 82.9 |
|  | Mpf | 0.006 | 6.800 | 0.996 | 0.821 | 0.008 | 11 | 0.071 | -0.014 | 6.780 | 0.886 | 0.007 | 5.0 | 918.8 |
|  | Total Samples | 0.190 | 6.149 | 0.859 | 0.592 | 0.002 | 64 | 0.436 | -0.014 | 5.838 | 0.734 | 0.001 | 30.1 | 980.5 |

**Table S4**. The parameters of m-DAR (metagenome diversity-area relationship) models fitted for MFGC (metagenome functional gene cluster) diversity, averaged from 100 times of re-sampling (GO)

| **Orders** | **Treatments** | **m-DAR PL (power law) Model** | | | | | | **m-DAR PLEC (power law with exponential cutoff) Model** | | | | | | |
| --- | --- | --- | --- | --- | --- | --- | --- | --- | --- | --- | --- | --- | --- | --- |
|  |  | ***z*** | **ln(*c*)** | *g* | *R* | ***p*-value** | ***N*** | ***z*** | ***d*** | **ln(*c*)** | *R* | ***p*-value** | ***A_max_*** | ***D_max_*** |
| *q*=0 | Mhm | 0.228 | 7.578 | 0.829 | 0.947 | 0.000 | 21 | 0.343 | -0.016 | 7.509 | 0.973 | 0.000 | 21.1 | 3683.2 |
|  | Mhf | 0.116 | 8.196 | 0.916 | 0.949 | 0.000 | 11 | 0.179 | -0.015 | 8.185 | 0.977 | 0.000 | 12.0 | 4676.4 |
|  | Mpm | 0.351 | 7.125 | 0.724 | 0.937 | 0.000 | 21 | 0.560 | -0.030 | 6.999 | 0.967 | 0.000 | 18.9 | 3250.0 |
|  | Mpf | 0.108 | 8.205 | 0.923 | 0.948 | 0.000 | 11 | 0.186 | -0.019 | 8.192 | 0.980 | 0.000 | 10.0 | 4602.1 |
|  | Total Samples | 0.150 | 7.911 | 0.890 | 0.847 | 0.000 | 64 | 0.301 | -0.008 | 7.691 | 0.921 | 0.000 | 37.1 | 4809.9 |
| *q*=1 | Mhm | 0.031 | 5.658 | 0.979 | 0.785 | 0.001 | 21 | 0.067 | -0.006 | 5.645 | 0.854 | 0.002 | 11.6 | 311.8 |
|  | Mhf | 0.024 | 5.654 | 0.983 | 0.771 | 0.012 | 11 | 0.038 | -0.004 | 5.660 | 0.874 | 0.010 | 8.8 | 300.4 |
|  | Mpm | -0.174 | 5.313 | 1.114 | 0.602 | 0.013 | 21 | -0.083 | -0.008 | 5.278 | 0.751 | 0.004 | -10.1 | NA |
|  | Mpf | 0.013 | 5.680 | 0.991 | 0.815 | 0.009 | 11 | 0.019 | -0.003 | 5.687 | 0.881 | 0.009 | 7.5 | 300.8 |
|  | Total Samples | 0.031 | 5.595 | 0.978 | 0.558 | 0.003 | 64 | 0.060 | -0.002 | 5.562 | 0.672 | 0.002 | 32.5 | 302.6 |
| *q*=2 | Mhm | 0.007 | 4.314 | 0.995 | 0.728 | 0.008 | 21 | 0.018 | -0.002 | 4.308 | 0.808 | 0.003 | 11.0 | 76.2 |
|  | Mhf | 0.003 | 4.317 | 0.998 | 0.808 | 0.008 | 11 | 0.007 | -0.001 | 4.320 | 0.880 | 0.008 | 5.0 | 75.5 |
|  | Mpm | -0.496 | 4.459 | 1.291 | 0.631 | 0.009 | 21 | -0.243 | -0.023 | 4.172 | 0.766 | 0.003 | -10.4 | NA |
|  | Mpf | -0.005 | 4.331 | 1.003 | 0.813 | 0.008 | 11 | 0.002 | -0.001 | 4.329 | 0.886 | 0.007 | 1.8 | 75.8 |
|  | Total Samples | 0.018 | 4.259 | 0.988 | 0.579 | 0.004 | 64 | 0.032 | -0.001 | 4.246 | 0.677 | 0.001 | 33.2 | 75.7 |
| *q*=3 | Mhm | 0.006 | 3.739 | 0.996 | 0.707 | 0.007 | 21 | 0.014 | -0.001 | 3.734 | 0.796 | 0.005 | 11.9 | 42.7 |
|  | Mhf | 0.000 | 3.759 | 1.000 | 0.792 | 0.010 | 11 | 0.002 | 0.000 | 3.760 | 0.880 | 0.007 | 3.7 | 43.0 |
|  | Mpm | -0.476 | 3.945 | 1.281 | 0.643 | 0.007 | 21 | -0.201 | -0.027 | 3.635 | 0.771 | 0.002 | -7.6 | NA |
|  | Mpf | -0.006 | 3.771 | 1.004 | 0.832 | 0.006 | 11 | -0.001 | -0.001 | 3.769 | 0.883 | 0.008 | -1.6 | NA |
|  | Total Samples | 0.015 | 3.704 | 0.989 | 0.593 | 0.003 | 64 | 0.029 | -0.001 | 3.690 | 0.690 | 0.001 | 33.0 | 43.1 |

**Table S5**. The parameters of m-DAR (metagenome diversity-area relationship) models fitted for MFGC (metagenome functional gene cluster) diversity, averaged from 100 times of re-sampling (KEGG)

| **Orders** | **Treatments** | **m-DAR PL (power law) Model** | | | | | | **m-DAR PLEC (power law with exponential cutoff) Model** | | | | | | |
| --- | --- | --- | --- | --- | --- | --- | --- | --- | --- | --- | --- | --- | --- | --- |
|  |  | ***z*** | **ln(*c*)** | *g* | *R* | ***p*-value** | ***N*** | ***z*** | ***d*** | **ln(*c*)** | *R* | ***p*-value** | ***A_max_*** | ***D_max_*** |
| *q*=0 | Mhm | 0.304 | 7.411 | 0.766 | 0.946 | 0.000 | 21 | 0.474 | -0.024 | 7.308 | 0.973 | 0.000 | 19.6 | 3812.5 |
|  | Mhf | 0.141 | 8.317 | 0.897 | 0.957 | 0.000 | 11 | 0.184 | -0.010 | 8.310 | 0.978 | 0.000 | 18.0 | 5753.6 |
|  | Mpm | 0.528 | 6.665 | 0.558 | 0.937 | 0.000 | 21 | 0.869 | -0.048 | 6.460 | 0.969 | 0.000 | 18.0 | 3302.9 |
|  | Mpf | 0.119 | 8.293 | 0.914 | 0.953 | 0.000 | 11 | 0.203 | -0.020 | 8.279 | 0.984 | 0.000 | 10.2 | 5149.4 |
|  | Total Samples | 0.213 | 7.877 | 0.841 | 0.853 | 0.000 | 64 | 0.403 | -0.010 | 7.600 | 0.914 | 0.000 | 39.5 | 5873.0 |
| *q*=1 | Mhm | 0.185 | 6.536 | 0.864 | 0.755 | 0.002 | 21 | 0.417 | -0.034 | 6.404 | 0.863 | 0.001 | 12.4 | 1139.6 |
|  | Mhf | 0.018 | 6.997 | 0.987 | 0.821 | 0.006 | 11 | 0.027 | -0.003 | 7.002 | 0.886 | 0.008 | 9.2 | 1135.8 |
|  | Mpm | 0.568 | 5.238 | 0.518 | 0.761 | 0.003 | 21 | 1.144 | -0.082 | 4.895 | 0.856 | 0.001 | 14.0 | 870.2 |
|  | Mpf | 0.040 | 6.926 | 0.972 | 0.849 | 0.005 | 11 | 0.061 | -0.007 | 6.941 | 0.894 | 0.007 | 9.1 | 1113.5 |
|  | Total Samples | 0.109 | 6.644 | 0.921 | 0.613 | 0.002 | 64 | 0.272 | -0.009 | 6.421 | 0.745 | 0.000 | 29.8 | 1178.2 |
| *q*=2 | Mhm | 0.369 | 5.159 | 0.708 | 0.715 | 0.004 | 21 | 0.610 | -0.045 | 5.162 | 0.796 | 0.002 | 13.7 | 468.3 |
|  | Mhf | 0.026 | 6.281 | 0.982 | 0.815 | 0.007 | 11 | 0.047 | -0.006 | 6.284 | 0.874 | 0.008 | 7.5 | 562.2 |
|  | Mpm | 0.608 | 2.884 | 0.475 | 0.744 | 0.003 | 21 | 0.909 | -0.056 | 2.922 | 0.817 | 0.003 | 16.3 | 94.6 |
|  | Mpf | 0.040 | 6.238 | 0.972 | 0.805 | 0.010 | 11 | 0.069 | -0.009 | 6.252 | 0.884 | 0.007 | 8.1 | 559.6 |
|  | Total Samples | 0.175 | 5.716 | 0.871 | 0.594 | 0.001 | 64 | 0.445 | -0.015 | 5.335 | 0.724 | 0.000 | 29.9 | 602.7 |
| *q*=3 | Mhm | 0.348 | 4.473 | 0.727 | 0.707 | 0.006 | 21 | 0.561 | -0.038 | 4.464 | 0.797 | 0.003 | 14.6 | 223.5 |
|  | Mhf | 0.041 | 5.675 | 0.971 | 0.843 | 0.005 | 11 | 0.065 | -0.009 | 5.693 | 0.874 | 0.010 | 7.3 | 316.4 |
|  | Mpm | 0.452 | 2.293 | 0.632 | 0.735 | 0.004 | 21 | 0.722 | -0.048 | 2.297 | 0.812 | 0.003 | 15.0 | 34.1 |
|  | Mpf | 0.047 | 5.650 | 0.967 | 0.791 | 0.012 | 11 | 0.085 | -0.013 | 5.680 | 0.877 | 0.009 | 6.5 | 315.7 |
|  | Total Samples | 0.199 | 5.053 | 0.852 | 0.588 | 0.001 | 64 | 0.492 | -0.016 | 4.651 | 0.725 | 0.000 | 29.9 | 340.8 |

**Table S6**. The parameters of m-DAR (metagenome diversity-area relationship) models fitted for MFGC (metagenome functional gene cluster) diversity, averaged from 100 times of re-sampling (Nr)

| **Orders** | **Treatments** | **m-DAR PL (power law) Model** | | | | | | **m-DAR PLEC (power law with exponential cutoff) Model** | | | | | | |
| --- | --- | --- | --- | --- | --- | --- | --- | --- | --- | --- | --- | --- | --- | --- |
|  |  | ***z*** | **ln(*c*)** | *g* | *R* | ***p*-value** | ***N*** | ***z*** | ***d*** | **ln(*c*)** | *R* | ***p*-value** | ***A_max_*** | ***D_max_*** |
| *q*=0 | Mhm | 0.772 | 9.094 | 0.292 | 0.958 | 0.000 | 21 | 1.109 | -0.048 | 8.891 | 0.978 | 0.000 | 23.3 | 78484.8 |
|  | Mhf | 0.415 | 11.498 | 0.666 | 0.975 | 0.000 | 11 | 0.614 | -0.047 | 11.465 | 0.991 | 0.000 | 13.0 | 249574.1 |
|  | Mpm | 0.963 | 7.683 | 0.050 | 0.954 | 0.000 | 21 | 1.232 | -0.038 | 7.522 | 0.973 | 0.000 | 32.4 | 39125.7 |
|  | Mpf | 0.456 | 11.317 | 0.628 | 0.978 | 0.000 | 11 | 0.647 | -0.045 | 11.284 | 0.992 | 0.000 | 14.3 | 232692.5 |
|  | Total Samples | 0.489 | 10.672 | 0.597 | 0.886 | 0.000 | 64 | 0.861 | -0.020 | 10.129 | 0.936 | 0.000 | 43.0 | 270422.8 |
| *q*=1 | Mhm | 0.556 | 7.452 | 0.530 | 0.828 | 0.001 | 21 | 1.013 | -0.066 | 7.202 | 0.898 | 0.000 | 15.3 | 7714.2 |
|  | Mhf | 0.375 | 8.859 | 0.703 | 0.859 | 0.004 | 11 | 0.504 | -0.029 | 8.826 | 0.914 | 0.004 | 17.3 | 17306.4 |
|  | Mpm | 0.624 | 4.532 | 0.458 | 0.676 | 0.007 | 21 | 0.863 | -0.053 | 4.668 | 0.783 | 0.003 | 16.2 | 497.5 |
|  | Mpf | 0.332 | 8.698 | 0.741 | 0.872 | 0.004 | 11 | 0.342 | -0.004 | 8.708 | 0.920 | 0.003 | 90.6 | 20112.1 |
|  | Total Samples | 0.408 | 8.135 | 0.673 | 0.818 | 0.000 | 64 | 0.750 | -0.018 | 7.636 | 0.889 | 0.000 | 40.8 | 15772.3 |
| *q*=2 | Mhm | 0.200 | 4.397 | 0.851 | 0.726 | 0.005 | 21 | 0.401 | -0.032 | 4.324 | 0.808 | 0.003 | 12.4 | 138.8 |
|  | Mhf | 0.139 | 5.252 | 0.899 | 0.837 | 0.005 | 11 | 0.156 | -0.011 | 5.317 | 0.892 | 0.006 | 14.4 | 264.4 |
|  | Mpm | 0.110 | 2.756 | 0.921 | 0.686 | 0.008 | 21 | 0.057 | -0.002 | 2.932 | 0.776 | 0.005 | 35.7 | 21.7 |
|  | Mpf | 0.068 | 5.377 | 0.952 | 0.829 | 0.006 | 11 | 0.020 | 0.008 | 5.408 | 0.873 | 0.009 | -2.3 | NA |
|  | Total Samples | 0.169 | 4.967 | 0.876 | 0.606 | 0.003 | 64 | 0.387 | -0.013 | 4.687 | 0.739 | 0.001 | 30.8 | 277.5 |
| *q*=3 | Mhm | 0.145 | 3.545 | 0.894 | 0.714 | 0.006 | 21 | 0.293 | -0.024 | 3.495 | 0.802 | 0.003 | 12.3 | 51.3 |
|  | Mhf | 0.016 | 4.245 | 0.989 | 0.842 | 0.005 | 11 | 0.044 | -0.005 | 4.237 | 0.893 | 0.006 | 8.4 | 72.7 |
|  | Mpm | 0.092 | 2.297 | 0.934 | 0.672 | 0.008 | 21 | -0.019 | 0.010 | 2.450 | 0.773 | 0.004 | 1.9 | 11.7 |
|  | Mpf | -0.010 | 4.259 | 1.007 | 0.796 | 0.012 | 11 | -0.084 | 0.018 | 4.264 | 0.873 | 0.009 | 4.7 | 67.8 |
|  | Total Samples | 0.092 | 3.949 | 0.934 | 0.609 | 0.002 | 64 | 0.251 | -0.009 | 3.740 | 0.744 | 0.000 | 28.0 | 75.7 |

**Table S7**. The parameters of m-DAR (metagenome diversity-area relationship) models fitted for MFGC (metagenome functional gene cluster) diversity, averaged from 100 times of re-sampling (Swissprot)

| **Orders** | **Treatments** | **m-DAR PL (power law) Model** | | | | | | **m-DAR PLEC (power law with exponential cutoff) Model** | | | | | | |
| --- | --- | --- | --- | --- | --- | --- | --- | --- | --- | --- | --- | --- | --- | --- |
|  |  | ***z*** | **ln(*c*)** | *g* | *R* | ***p*-value** | ***N*** | ***z*** | ***d*** | **ln(*c*)** | *R* | ***p*-value** | ***A_max_*** | ***D_max_*** |
| *q*=0 | Mhm | 0.569 | 7.790 | 0.517 | 0.961 | 0.000 | 21 | 0.775 | -0.029 | 7.666 | 0.979 | 0.000 | 26.5 | 12464.3 |
|  | Mhf | 0.336 | 9.636 | 0.738 | 0.980 | 0.000 | 11 | 0.440 | -0.025 | 9.619 | 0.991 | 0.000 | 17.9 | 34456.1 |
|  | Mpm | 0.828 | 6.623 | 0.225 | 0.955 | 0.000 | 21 | 1.182 | -0.050 | 6.409 | 0.975 | 0.000 | 23.5 | 7787.9 |
|  | Mpf | 0.343 | 9.569 | 0.732 | 0.975 | 0.000 | 11 | 0.486 | -0.034 | 9.544 | 0.990 | 0.000 | 14.4 | 31331.4 |
|  | Total Samples | 0.445 | 8.807 | 0.638 | 0.884 | 0.000 | 64 | 0.765 | -0.017 | 8.342 | 0.933 | 0.000 | 44.6 | 35602.0 |
| *q*=1 | Mhm | 0.265 | 7.355 | 0.799 | 0.910 | 0.000 | 21 | 0.503 | -0.034 | 7.212 | 0.959 | 0.000 | 14.9 | 3190.1 |
|  | Mhf | 0.168 | 7.774 | 0.877 | 0.824 | 0.009 | 11 | 0.223 | -0.017 | 7.792 | 0.899 | 0.006 | 13.5 | 3461.0 |
|  | Mpm | 0.778 | 5.356 | 0.286 | 0.743 | 0.004 | 21 | 0.965 | -0.045 | 5.473 | 0.823 | 0.004 | 21.3 | 1732.6 |
|  | Mpf | 0.127 | 7.741 | 0.908 | 0.829 | 0.008 | 11 | 0.164 | -0.014 | 7.771 | 0.890 | 0.008 | 12.0 | 3026.2 |
|  | Total Samples | 0.184 | 7.437 | 0.864 | 0.681 | 0.002 | 64 | 0.379 | -0.011 | 7.156 | 0.790 | 0.000 | 35.8 | 3404.1 |
| *q*=2 | Mhm | 0.167 | 6.632 | 0.877 | 0.804 | 0.001 | 21 | 0.373 | -0.029 | 6.512 | 0.891 | 0.001 | 12.7 | 1196.0 |
|  | Mhf | 0.082 | 6.938 | 0.942 | 0.828 | 0.006 | 11 | 0.118 | -0.012 | 6.952 | 0.894 | 0.005 | 10.0 | 1219.2 |
|  | Mpm | -0.338 | 5.424 | 1.209 | 0.642 | 0.009 | 21 | 0.340 | -0.081 | 4.977 | 0.762 | 0.005 | 4.2 | 167.9 |
|  | Mpf | 0.049 | 6.851 | 0.966 | 0.808 | 0.009 | 11 | 0.061 | -0.005 | 6.862 | 0.897 | 0.005 | 12.4 | 1048.2 |
|  | Total Samples | 0.171 | 6.433 | 0.874 | 0.630 | 0.001 | 64 | 0.346 | -0.010 | 6.213 | 0.712 | 0.001 | 33.5 | 1191.2 |
| *q*=3 | Mhm | 0.151 | 5.945 | 0.890 | 0.709 | 0.006 | 21 | 0.337 | -0.027 | 5.850 | 0.819 | 0.003 | 12.3 | 577.5 |
|  | Mhf | 0.077 | 6.217 | 0.945 | 0.807 | 0.009 | 11 | 0.076 | -0.004 | 6.253 | 0.879 | 0.008 | 17.9 | 599.1 |
|  | Mpm | -0.535 | 5.070 | 1.310 | 0.645 | 0.010 | 21 | 0.277 | -0.093 | 4.363 | 0.767 | 0.005 | 3.0 | 80.6 |
|  | Mpf | 0.015 | 6.178 | 0.990 | 0.797 | 0.010 | 11 | -0.025 | 0.007 | 6.201 | 0.883 | 0.008 | 3.7 | 489.2 |
|  | Total Samples | 0.158 | 5.739 | 0.884 | 0.593 | 0.002 | 64 | 0.328 | -0.010 | 5.538 | 0.684 | 0.001 | 32.2 | 571.5 |
